# Supplementary material for: Preferences for oral PrEP dosing among adolescent boys and young men in three sub-Saharan African countries
Source: PLoS One. 2023 Oct 9;18(10):e0285132. doi: 10.1371/journal.pone.0285132 (PMC10561834; doi:10.1371/journal.pone.0285132)
Supplement: Attachment — Submitted filename: Response to Reviewers.docx [file pone.0285132.s001.docx]

06^th^	Feb 2023

Dear Editor,

Re: **Response to the reviewers’ comments:** Ref. No:  EMID: a742d9a903b90479

Title:

**PrEP preference among adolescent boys and young men in three sub-Saharan African countries**

Thank you very much for reviewing our manuscript and providing us the opportunity to respond to the reviewers’ comments. Please see below our responses to the issues raised by the reviewers.

Yours sincerely,

Ayoub Kakande

Submitting Author

On behalf of the CHAPS consortium

Review Comments to the Author

Reviewer #1: 1. ETHICAL CONSIDERATIONS: Why was the consent not sought for the younger boys? Was assent sought?

Response: “Parental waiver of consent was approved by ethical boards for use in Cape Town, Entebbe, and Chitungwiza. Written study informed consent or assent was obtained from all participants”.

Prior to study participation, written informed consent was obtained from participants 18 years and older. Parental consent and participant assent were also required for participants 17 years and younger. Sites in Uganda, Zimbabwe, and Cape Town, South Africa had parental consent waivers in place according to their respective guidelines. Participants were reimbursed for their time and participation based on site national requirements and guidelines

.

2. The authors can give slightly more contextual background information about the adolescents from the 3 different countries...to give more insight in the study findings

Response: In sub-Saharan Africa, the overall prevalence of ‘any substance use’ is estimated at 41.6% with caffeine containing products at 41.2% and alcohol at 32.8% calling for interventions that are comprehensive and targeted at adolescents these settings(6). Many adolescents are at risk of HIV because

Purposive community-based sampling was used to recruit young people aged 13-24 years in four settings: Johannesburg and Cape Town in South Africa, Wakiso in Uganda, and Chitungwiza in Zimbabwe. In South Africa and Zimbabwe, participants were recruited from community groups, schools, churches whose HIV prevalence is 15% amongst 15-49 year olds, 24% , 20% amongst 15-24 year olds and 7.3% amongst 20-24 respectively(18), bars, taxi ranks, and other public meeting places, of multiple partnerships and insufficient condom(7).

3. Purposive community-based sampling data collection methodology worked well for the study but replication of the study design is limited.

Response: Thanks for the comments. We acknowledge the difficulty with replication of a study that used a non-probability sampling method such as purposive sampling. We have now included this as a limitation in the discussion section.

Reviewer #2: This manuscript explores preferences for on-demand vs. daily PrEP among adolescent boys and young men in SSA, a group at high risk of HIV infection. Overall, I found the qualitative data compelling and interesting. I think there are several issues regarding framing of on-demand PrEP and the quantitative analysis that need to be addressed. First, to the best of my knowledge, on-demand PrEP has been formally recommended by public health organizations for heterosexual males. This may not be true, but the authors do not provide citations supporting on-demand PrEP for heterosexual males. This is not a major limitation, but I think their study needs to be better contextualized in the Intro and Discussion as on-demand PrEP would be “off-label” for most participants, and has only been studied with TDF-FTC, not TAF-FTC. Unless on-demand PrEP has been formally recommended for heterosexual men, I think conclusions from this study need to be better contextualized. Second, I think the multivariable model needs to be clarified, which variables were included in the final adjusted model? The quantitative results are confusing as well, when the one group found to be significantly associated with preferring on-demand PrEP is “prefer not to answer,” I don’t know what to make of this finding. Third, I think greater detail to the mixed methods strategy is needed: was this a primarily explanatory model vs exploratory? It seems the data were obtained sequentially but analyzed simultaneously, but it is not clear.

1.Title: After reading the abstract, it becomes clear that the focus of the research is on-demand vs. daily PrEP. I would suggest including this focus in the title, since “PrEP preference” is a bit vague.

Response: Thank you for this suggestion. As there are only two oral PrEP choices i.e. on-demand and daily we felt “PrEP preference” was appropriate and prefer retaining the title as is since the investigation was not driven by choosing between on demand oral PrEP and daily oral PrEP but a participant choosing their PrEP delivery mode of convenience.

2.Abstract: Authors should specify mixed methods methodology briefly in abstract: was the process sequential (first quantitative, then qualitative?) or simultaneous? Exploratory vs. explanatory? I would also mention that these data came from a larger study… did the social science study precede the RCT?

Response: The abstract has been reworded to “A cross-sectional sequential exploratory mixed-methods study amongst males aged 13–24 years was conducted between April and September 2019 as part of the CHAPS trial. Group discussions (GDs) and In-Depth Interviews (IDIs) focused on motivations and hindrances for HIV testing, PrEP preference, and reasons for the uptake of PrEP. A thematic approach was used to analyse the qualitative data. A quantitative survey following the qualitative work covered questions on demographics, HIV risk and PrEP preferences (on-demand vs. daily). For quantitative analysis, we fitted logistic regression models to determine factors associated with on-demand vs daily PrEP preference.”

The data did not come from a larger study. A separate phase 2b Pharmacokinetic dose finding for insertive sex followed this study utilising findings to facilitate design and implementation of the trial.

3. Abstract: The factor “advanced planning of sex” needs to be clarified in the Results. This is confusing since the group is “prefer not to answer.”

Response: Thank you for this suggestion. We have now replaced this and instead show the adjusted odds ratio comparing men who knew they would have sex >24 hours in advance versus mean who knew they would have sex <2 hours in advance, which is 1.4 (0.9-2.3). There was a general pattern of increasing odds of preferring on demand PrEP as the amount of time with which sex was planned increased.
4. Introduction, third paragraph: Since long acting injectable PrEP is now available in many regions, the authors should clarify PrEP as oral PrEP. The IPERGAY study also only considered TDF/FTC.

Response: This has been clarified in the paper “On-demand oral PrEP involves taking PrEP around the time of sexual activity (two doses taken 24 hours before sex followed by single doses for the two days after sex) and has been shown to be highly effective in men who have sex with men (MSM) (7). However, the widespread use of on-demand oral PrEP is hindered by a lack of data in other populations and concerns regarding regime complexity (9).”.

5. Introduction, third paragraph, last sentence: this sentence and the citations are misleading. On-demand PrEP is not recommended in that citation provided (from 2015); I believe it was not recommended by the WHO formally until 2019, and even so, was done with caveats: only for men who have sex with men. While I don’t think this is a major weakness of the paper, this caveat needs to be recognized earlier (the authors do mention in the third paragraph, but I think it needs to be earlier), particularly since the focus of this paper is on heterosexual adolescent boys and young men.

Response: This has been edited to read “however, the widespread use of on-demand oral PrEP is hindered by a lack of data in other populations and concerns regarding regime complexity (9). Even though the WHO recommends r oral PrEP in populations with substantial risk such as MSM (site), other populations such as adolescents and young people are not catered for , suggesting a lack of policies,”

6. Methods, Data collection: Would re phrase “reasons for the uptake of PrEP uptake.”

Response: This has been edited to read: “The IDIs and GDs generally focused on motivations and hindrances for HIV testing, PrEP preference (daily vs on-demand), and reasons for the PrEP preference”

7.Methods: Did the authors collect data on number of partners? Or if they had a regular partner?

Response: Data were collected on the number of partners. Specifically, participants were asked, “In the past 6 months, how many different people have you had sex with?” however we did not include it in analysis

8.Methods, Data Analysis: The authors state that the main outcome was analysed as a binary variable. Was it also collected as binary, or were participants given a Likert scale of preference for on-demand vs. daily PrEP?

Response: Thank you for this comment, data on the main outcome were collected as a binary variable (participants chose between on-demand and daily PrEP).

9. Methods: Additional detail on study measures would be helpful. For instance, how was the measure of “hours of premeditation before sex” phrased?

Response: Considering the most recent sexual encounter, a participant was asked to estimate how far ahead he was likely to have sex (premeditation about sex). This was collected as a categorical variable (<2 hrs, 2-12 hrs, 13-24hrs, >24hrs, prefer not to answer). More details on how this was phrased have been provided in the data analysis section of the manuscript. The timelines 2-24hours aligned with dosing recommendations for on demand PrEP (9)

10.Methods: The authors should include more details on the type of mixed methodology used, e.g., sequential vs. simultaneous? Exploratory?

Response: “This study was part of tan HIV prevention programme called “The Combined HIV Adolescent Prevention Study (CHAPS), a joint African-European HIV prevention collaboration running in South Africa, Uganda, and Zimbabwe, focusing on young adults (13-24 years old) between April and November 2019. CHAPS comprised of two primary studies running sequentially: (i) Firstly, a social science mixed methods study investigating the acceptability and feasibility of taking daily and on-demand PrEP (14), and (ii) a Phase II randomized clinical trial investigating the optimum on-demand PrEP dosing schedule for insertive sex for both FTC-TDF and FTC-TAF (ClinicalTrials.gov [NCT03986970](https://clinicaltrials.gov/ct2/show/NCT03986970), June 2019). Details of the CHAPS study have been previously described (15). We used a cross-sectional sequential exploratory mixed-methods analysis of the data drawn from the CHAPS social science study.”

11.Methods/Results: The authors chose a backward elimination model, yet the results in Table 2 are a little confusing. Some factors in the bivariate model (frequency of sex, heads household, number of people in the household) appear significant at the bivariate model, but are not included in the final model. It is also unclear whether age was significant in the adjusted model, since the 95% Cis cross 1, but the p-value is 0.002.

Response: At bivariable analysis, variables that had a likelihood ratio test p-value of <0.150 for inclusion in the model (not Wald p-value for individual categories of the variable) were considered for multivariable analysis. The multivariable model was built basing on likelihood ratio test (LRT) p-value i.e. LRT p-value of addition into the model of a given factor. If a factor had a model p-value <0.05 at bivariable analysis but its LRT p-value for inclusion into the multivariable model was >0.05, that factor was excluded.

The p=0.002 for age is the LRT p-value for inclusion of age factor into the multivariable model and not Wald p-value for comparisons of each individual category with the reference (baseline) category that can be related directly to the 95%CI.

12.Results: The authors state that “odds of preference for on-demand PrEP increased with increased hours of premeditation about sex” … this is only true in the unadjusted model, correct? In addition, the relationship does not appear linear. I also do not know what to make of the “prefer not to answer” being significant… since this could be very little time, or a lot of time.

Response: We thank the reviewer for this observation. We have corrected this statement to include only statistically significant results about hours of premeditation before sex. More details were provided in the data analysis section of the manuscript. We have also updated the abstract to show the OR comparing >24 hours advance planning with <2 hours advance planning.

13. Results, Qualitative (page 15-16) I would suggest retitling point 3) since many of the quotes deal with tablet fatigue, rather than side effects. These are related but not quite the same.

Response: Thank you this has been retitled to “Fewer tablets meant lower pill burden or less pill fatigue”

14. Results: Would suggest moving Table 3 higher up in the Results, in the beginning of the qualitative themes.
Response: This has been moved before the themes.

15. Discussion: Some mention of long acting injectable deserves mention, perhaps as a limitation of the study if not discussed.

Response: Another limitation is that even though long-acting injectable Cabotegravir may be offered as an additional prevention choice for people at substantial risk of HIV infection, as part of combination prevention approaches (22) in this study we did not provide it as a choice for the adolescents because it was only approved in late 2022, and was yet to be rolled out in healthcare facilities.

16.Conclusions: the authors state “following WHO support for on-demand PrEP for all men, not just MSM…” I am not sure this is correct, and the authors have not cited WHO literature supporting on-demand PrEP for heterosexual men.

In the study, we found high acceptance of on demand PrEP among adolescent and young heterosexual men, particularly in Uganda and Zimbabwe. Following WHO recommendation for on-demand (event-driven) oral PrEP for MSM(12),Our findings present an opportunity to engage regional policy makers and WHO about including on-demand strategies for young heterosexual men in PrEP programming similar to MSMs (12) and provide some insights into their HIV prevention decision making.

We hope that given our results, stakeholders can take into account that there is high user acceptability of this form of HIV prevention.

DOI

[https://doi.org/10.17037/DATA.00003232](https://protect-eu.mimecast.com/s/mzhECKQDxUZDqoYSM2cXn?domain=doi.org)
