# Supplementary material for: Preferences for oral PrEP dosing among adolescent boys and young men in three sub-Saharan African countries
Source: PLoS One. 2023 Oct 9;18(10):e0285132. doi: 10.1371/journal.pone.0285132 (PMC10561834; doi:10.1371/journal.pone.0285132)
Supplement: Attachment — Submitted filename: Response to Reviewers.docx [file pone.0285132.s002.docx]

20th	Mar 2023

Dear Editor,

Re: **Response to the reviewers’ comments:** Ref. No:  [PONE-D-22-28042R1] - [EMID:f381a81dd41eee53]

Title:

**PrEP preference among adolescent boys and young men in three sub-Saharan African countries**

Thank you very much for reviewing our manuscript and providing us the opportunity to respond to the reviewers’ comments. Please see below our responses to the issues raised by the reviewers.

Yours sincerely,

Ayoub Kakande

Submitting Author

On behalf of the CHAPS consortium

6. Review Comments to the Author

Title: While I appreciate the author’s comment on why they retained the title, perhaps I need to clarify why I think “PrEP preference” is too vague. This reviewer believes it is imperative to clarify in the title what the authors are investigating, given the large literature on PrEP preferences. PrEP preference could mean mode of delivery (e.g., oral vs. injectable vs. implantable), rather than oral dosing schedule. Even if injectable is not approved yet in this region, since this manuscript only assesses “preferences” (and not use), it is reasonable to assume that the authors intend to study injectable PrEP from the title alone. Another alternative “preference” would be site of delivery, e.g., clinic based, home delivery, pharmacy delivery, however, the authors do not investigate this. I believe further clarity in the title is needed, as both a reviewer and a potential reader of the manuscript would desire further clarification of WHAT is exactly being studied. A suggested title revision that would address my concern would be “Preferences for on-demand oral PrEP among…” or “Preferences for oral PrEP dosing”

Response: We thank the reviewer for this comment, we have edited the title to “Preferences for oral PrEP dosing among adolescent boys and young men in three sub-Saharan African countries”

Introduction, line 83: suggest clarifying in this sentence “Oral pre-exposure prophylaxis”
Introduction, line 93 (and elsewhere): suggest changing “regime” to “regimen”

Response: We thank the reviewer for this comment, we have edited pre-exposure prophylaxis to read **Oral** pre-exposure prophylaxis, and we have also edited all regimes to regimens

Results: If the number of partners was measured, I would include it in Table 1 as a descriptive characteristic. Reasonable to leave out of analysis.

Response: We thank the reviewer for this comment, we have added “number of sex partners in the last 6 months in table 1”

Data Analysis: Include the primary outcome in the data analysis section. I’m not sure why this was not included in the revision.

Response: We thank the reviewer for this comment, in the data analysis section we have included “Data on the main outcome were collected as a binary variable (participants chose between on-demand and daily oral PrEP).

Prior point about multivariable model building: The reviewer appreciates the authors explanation of how they chose to build the multivariable model, but is again confused why this explanation is not included in the revised manuscript.

Response: we thank the reviewer for this comment and acknoldge the omission, we have now included it as “At bivariable analysis, variables that had a likelihood ratio test p-value of <0.150 (not Wald p-value for individual categories of the variable) were considered for multivariable analysis. The multivariable model was built basing on likelihood ratio test (LRT) p-value i.e. LRT p-value of addition into the model of a given factor. If a factor had a model p-value <0.05 at bivariable analysis but its LRT p-value for inclusion into the multivariable model was >0.05, that factor was excluded.”

Table 3: Does the column reading “Less tables mean fewer side effects” need to also be revised to “fewer tables mean lower pill burden or less pill fatigue?” as revised elsewhere in the manuscript?

Response: We thank the reviewer for this comment, we have replaced “Less tablets mean fewer side effects” with “fewer tablets mean lower pill burden or less pill fatigue?
